# Effects of *Interleukin-10* Polymorphisms, *Helicobacter pylori* Infection, and Smoking on the Risk of Noncardia Gastric Cancer

**DOI:** 10.1371/journal.pone.0029643

**Published:** 2012-01-03

**Authors:** Jeongseon Kim, Young Ae Cho, Il Ju Choi, Yeon-Su Lee, Sook-Young Kim, Aesun Shin, Soo-Jeong Cho, Myeong-Cherl Kook, Ji Hyung Nam, Keun Won Ryu, Jun Ho Lee, Young-Woo Kim

**Affiliations:** 1 Cancer Epidemiology Branch, National Cancer Center, Goyang, Korea; 2 Center for Gastric Cancer, National Cancer Center, Goyang, Korea; 3 Functional Genomics Branch, National Cancer Center, Goyang, Korea; 4 Center for Cancer Prevention and Detection, Goyang, Korea; National Cancer Center, Japan

## Abstract

**Objective:**

Both variations in the interleukin-10 (IL10) gene and environmental factors are thought to influence inflammation and gastric carcinogenesis. Therefore, we investigated the associations between *IL10* polymorphisms, *Helicobacter pylori (H. pylori)* infection, and smoking in noncardia gastric carcinogenesis in Koreans.

**Methods:**

We genotyped three promoter polymorphisms (-1082A>G, -819T>C, and -592 A>C) of *IL10* in a case-control study of 495 noncardia gastric cancer patients and 495 sex- and age-matched healthy controls. Multiple logistic regression models were used to detect the effects of *IL10* polymorphisms, *H. pylori* infection, and smoking on the risk of gastric cancer, which was stratified by the histological type of gastric cancer.

**Results:**

The *IL10*-819C and -592C alleles were found to have complete linkage disequilibrium, and all three *IL10* polymorphisms were associated with an increased risk of intestinal-type noncardia gastric cancer. These associations were observed only in *H. pylori-*positive subjects and current smokers. A statistically significant interaction between the *IL10*-592 genotype and *H. pylori* infection on the risk of intestinal-type gastric cancer was observed (*P* for interaction  = 0.047). In addition, *H. pylori*-positive smokers who were carriers of either the *IL10*-1082G (OR [95% CI]  = 17.76 [6.17−51.06]) or the *-592C* (OR [95% CI]  = 8.37 [2.79−25.16]) allele had an increased risk of intestinal-type gastric cancer compared to *H. pylori-*negative nonsmokers homozygous for *IL10*-1082A and -592A, respectively. The interaction between the *IL10*-1082 polymorphism and the combined effects of *H. pylori* infection and smoking tended towards significance (*P* for interaction  = 0.080).

**Conclusions:**

Inflammation-related genetic variants may interact with *H. pylori* infection and smoking to increase the risk of noncardia gastric cancer, particularly the intestinal-type. These findings may be helpful in identifying individuals at an increased risk for developing noncardia gastric cancer.

## Introduction

Gastric cancer is the second most common cause of cancer mortality globally and the prevalence of gastric cancer is higher in Asians than in Western populations [1]. Numerous studies have supported the concept that gastric inflammation is a critical component of tumor development [2], [3]. *H. pylori* is a strong risk factor for gastric cancer, likely due to the extensive inflammation in the stomach mucosa caused by this bacteria [4]. However, considering that only a small portion of *H. pylori-*infected subjects eventually develop gastric cancer, the pathway from *H. pylori* infection to carcinogenesis may be affected by several factors, including *H. pylori* strain variability, lifestyle (e.g., smoking, low vegetable/fruit consumption, high salt intake), and host genetics [5], [6].

In chronic gastric inflammation, activated neutrophils and mononuclear cells produce different types of cytokines that are crucial in regulating inflammation [7], [8]. Altered cytokine levels have been observed during tumor initiation and promotion in the stomach [9], [10]. Interleukin-10 (IL10) is a multifunctional anti-inflammatory cytokine that downregulates cell-mediated immune responses and cytotoxic inflammatory responses. Three polymorphic promoter variants, located at positions -1082 (A>G), -819 (T>C), and -592 (A>C), have been identified in the *IL10* gene. It has been reported that these variants of *IL10* are associated with increased IL10 production [11]–[13] and an elevated risk of gastric cancer [14], [15]. Furthermore, environmental factors, such as cigarette smoking, may alter cytokine expression in a manner more favorable for the development of gastric inflammation and carcinogenesis [16]. Therefore, inflammation-related polymorphisms may interact with environmental factors in the development of gastric cancer.

Although many studies have investigated the roles of *IL10* promoter polymorphisms and the risk of gastric cancer, the findings remain inconclusive, possibly due to differences in study design and/or ethnic differences in the populations studied [14], [15], [17]–[19]. Furthermore, gastric cancers that differ in anatomic location and/or histological type have different clinicopathological characteristics [3], [6]. *H. pylori* infection and chronic inflammation have been more closely linked with noncardia gastric cancer than with cardia gastric cancer and may induce the histological progression to gastric cancer [3]. In addition, the impact of genetic and environmental factors may differ by the histological type of gastric cancer [6]. Therefore, the present study aimed to investigate the effects of *IL10* genetic variants, *H. pylori* infection, cigarette smoking, and their interactions on the risk of noncardia gastric carcinogenesis. We also examined the differential effects of these factors according to the histological type of gastric cancer.

## Materials and Methods

### Study Population

This study was a hospital-based case-control study. A total of 797 patients with noncardia gastric cancer were recruited from the National Cancer Center Hospital in Goyang between 2003 and 2007. Only histologically-confirmed adenocarcinoma patients were included in this study, and other types of neoplasms found in the stomach (e.g., MALT lymphomas, other types of primary gastric lymphoma, gastrointestinal stromal tumors, carcinoid tumors, or adenomas) were excluded. Nine hundred thirty healthy controls who underwent upper endoscopy at the same institution for gastric cancer screening in the National Cancer Screening Program in Korea and were without significant gastrointestinal symptoms were recruited in 2007. All cases and controls were of Korean ancestry. For both gastric cancer cases and controls, we excluded subjects who had a history of other malignant neoplasms, had a history of previous *H. pylori* treatment, had not received an *H. pylori* evaluation, or were lacking in information about their genetic background.

Cases and controls were frequency-matched by age (within 5-years) and sex. In total, 495 cases and 495 controls were used for the final analyses. Noncardia gastric cancer patients were classified into two subgroups (intestinal-type and diffuse-type) based on the histopathology according to the Lauren classification [20]. The clinical information and genetic materials were obtained under the approval of the Institutional Review Board of the National Cancer Center in Korea, and written informed consent was obtained from each subject.

### Data Collection

The questionnaire data and blood samples were obtained at the initial recruitment of both cases and controls. All participants were asked to complete a self-administered questionnaire about their sociodemographic characteristics (e.g., age, family history of gastric cancer, income, and education), smoking habits, alcohol intake, and personal medical history. *H. pylori* infection status was determined by a rapid urease test and a histological evaluation. The rapid urease test was performed according to the manufacturer's instructions (Pronto Dry. Medical Instruments Corporation, Solothurn, Switzerland). For the histological evaluation of *H. pylori,* biopsy specimens were obtained from three different areas, the gastric antrum, the corpus lesser curvature, and the corpus greater curvature, to increase the sensitivity of the procedure. Two pieces of biopsy specimens were obtained from each site, and Wright-Giemsa staining was performed on the biopsy samples to determine if *H. pylori* was present.

Genotyping of the *IL10* promoter (-1082/-819/-592) was conducted and interpreted by a researcher blinded to case/control status. Briefly, genomic DNA was prepared from peripheral blood samples with an automatic DNA extraction system (BioRobot M48 Workstation, Qiagen, Inc., Valencia, CA) following the manufacturer's instructions, and the purity and concentration of isolated DNA were determined using picogreen (Molecular Probes, Inc., Eugene, OR). A total of 10 ng per sample was used for genotype analysis. Genotyping for the three SNPs was performed by the iPLEX Gold assay (Sequenom, San Diego, CA), which is based on MALDI-TOF spectrometry, per the manufacturer's protocol, and the resulting genotype data were collected by Typer v4.0 (Sequenom). To ensure quality control, subsets of the samples were run as duplicates and 5% of the wells in a 384-well plate were used as negative controls to monitor for contamination. For all results, genotype clusters were examined manually for their fitness.

### Statistical Analysis

The demographic characteristics, environmental factors, and gene frequencies of *IL10* in patients and controls were compared using χ^2^ statistics. The Hardy-Weinberg equilibrium of genotype frequencies in the controls was tested with the χ^2^ test. The association between *IL10* genetic variants (-1082/-819/-592) and the risk of gastric cancer was estimated by multivariate logistic regression with adjustments for age, sex, *H. pylori* infection, smoking, alcohol consumption, income, and education and was expressed as odds ratios (ORs) with 95% confidence intervals (CIs). Haplotype analyses were also conducted. *IL10* haplotypes were constructed based on the frequencies of three SNPs (-1082/-819/-592) in this study population. These polymorphisms were found to be in strong linkage disequilibrium, and three of the eight possible haplotypes (GCC, ACC, ATA) segregate in the Korean population. We defined the more common homozygous genotype/haplotype in the control subjects as the reference genotype/haplotype. The effects of *H. pylori* infection and smoking on the association between genetic variants and the risk of gastric cancer were examined in a subgroup analysis by *H. pylori* infection status (*H. pylori*-positive/*H. pylori*-negative) or smoking status (non-smoker/past smoker/current smoker). In addition, to investigate the combined effects of *H. pylori* infection and smoking on the association between genetic variants and the risk of gastric cancer, subjects were grouped according to their dichotomized *H. pylori* infection (*H. pylori*-positive/*H. pylori*-negative) and smoking (smokers (all subjects who had ever smoked were classified as smokers)/non-smokers) status. Data were stratified by the histological type of gastric cancer (intestinal-type/diffuse-type).The significance of the interactions between genetic variants and environmental risk factors was assessed with the likelihood ratio test, which compared the model with the interaction term with the one that only contained the main effects. All statistical analyses were performed with SAS 9.1 software (SAS Institute Inc. Cary, NC). Two-sided *P*-values less than 0.05 were regarded as statistically significant.

## Results

The demographics of the study subjects are shown in Table 1. No differences between cases and controls in the distribution of age, sex, and family history of gastric cancer were found. However, cases were more likely to have *H. pylori* infection (*P*<0.001), more likely to smoke (*P*<0.001), and less likely to drink alcoholic beverages (*P* = 0.027) than controls. In addition, cases also had a lower mean income (*P* = 0.017) and less education (*P*<0.001) than controls. With regard to genetic frequencies, no significant differences between cases and controls in the frequencies of the *IL10*-1082/-819/-592 polymorphisms were present. *IL10-*819 and *IL10*-592 were found to be in complete linkage disequilibrium.

**Table 1:** Characteristics of the Study Subjects*.

	Gastric Cancer Cases (n = 495)	Controls (n = 495)	*P*-value
Age (years), mean ± SD	54.9±8.4	54.3±7.4	0.208
Male	337 (68.1)	337 (68.1)	1.000
Family history of gastric cancer	220 (44.4)	225 (45.5)	0.749
*H. pylori* infection (positive)	443 (89.9)	329 (66.6)	<0.001
Smoking status			
Non-smoker	183 (37.0)	208 (42.0)	<0.001
Past smoker	108 (21.8)	158 (31.9)	
Current smoker	204 (41.2)	129 (26.1)	
Alcohol consumption			
Non-drinker	167 (33.8)	147 (29.7)	0.027
Past drinker	50 (10.1)	33 (6.7)	
Current drinker	277 (56.1)	315 (63.6)	
Income			
Low	205 (41.4)	165 (33.3)	0.017
Medium	242 (48.9)	286 (57.8)	
High	48 (9.7)	44 (8.9)	
Education (years)			
<12	231 (46.7)	145 (29.3)	<0.001
≥12	264 (53.3)	350 (70.7)	
*IL10*-1082			
AA	416 (84.0)	435 (87.9)	0.198
AG	72 (14.6)	56 (11.3)	
GG	7 (1.4)	4 (0.8)	
*IL10*-819			
TT	231 (46.7)	248 (50.1)	0.325
CT	214 (43.2)	191 (38.6)	
CC	50 (10.1)	56 (11.3)	
*IL10*-592			
AA	231 (46.7)	248 (50.1)	0.325
CA	214 (43.2)	191 (38.6)	
CC	50 (10.1)	56 (11.3)	
Histological type of gastric cancer			
Intestinal	253 (51.1)	-	
Diffuse	214 (43.2)	-	
Mixed	28 (5.7)	-	
Stage of gastric cancer			
Early gastric cancer†	279 (56.4)	-	
Advanced gastric cancer‡	216 (43.6)	-	

*Results presented as n (%) unless otherwise indicated.

† Depth of tumor invasion limited to mucosa or submucosa.

‡ Depth of tumor invasion involved the proper muscle layer or beyond.

To determine whether genetic susceptibility to noncardia gastric cancer was present, the association between *IL10* variants and the risk of noncardia gastric cancer stratified by histological type was investigated (Table 2). The results of a multivariate regression model revealed that *IL10-*1082G (OR [95% CI]  = 1.69 [1.06−2.69]) and -592C (OR [95% CI]  = 1.55 [1.10−2.18]) carriers had a higher risk of intestinal-type gastric cancer than subjects homozygous for the *IL10-*1082A allele and -592A allele, respectively. In addition, carriers of the GCC haplotype had an increased risk of intestinal-type gastric cancer compared to ATA haplotype carriers (OR [95% CI]  = 1.64 [1.06−2.54]). We also examined the association between *IL10* genetic variants and the risk of gastric cancer after stratifying noncardia gastric cancer by stage (data not shown). However, we did not find any differences in the risk of gastric cancer between the disease stages.

**Table 2:** The Association between *IL10* Genetic Variants and the Risk of Gastric Cancer, Stratified by Histological Type of Noncardia Gastric Cancer.

		All type			Intestinal type (n = 253)			Diffuse type (n = 214)		
	No. of Controls (%)	No. of Cases (%)	Crude OR (95% CI)	Multivariate OR (95% CI)*	No. of Cases (%)	Crude OR (95% CI)	Multivariate OR (95% CI)*	No. of Cases (%)	Crude OR (95% CI)	Multivariate OR (95% CI)*
***IL10*** **-1082**										
AA	435 (87.9)	416 (84.0)	1.0	1.0	209 (82.6)	1.0	1.0	182 (85.1)	1.0	1.0
G carrier	60 (12.1)	79 (16.0)	1.37 (0.92, 1.99)	1.42 (0.96, 2.10)	44 (17.4)	1.59 (1.01, 2.51)	1.69 (1.06, 2.69)	32 (14.9)	1.30 (0.80, 2.03)	1.18 (0.71, 1.96)
***IL10*** **-592** †										
AA	248 (50.1)	231 (46.7)	1.0 (Ref)	1.0	107 (42.29)	1.0	1.0	102 (47.7)	1.0	1.0
C carrier	247 (49.9)	264 (53.3)	1.15 (0.89, 1.47)	1.20 (0.91, 1.57)	146 (57.71)	1.37 (1.01, 1.86)	1.55(1.10, 2.18)	112 (52.3)	1.10 (0.80, 1.52)	1.18 (0.83, 1.67)
**Haplotype** ‡										
ATA	687 (69.4)	676 (68.3)	1.0 (Ref)	1.0 (Ref)	332 (65.6)	1.0 (Ref)	1.0 (Ref)	294 (68.7)	1.0 (Ref)	1.0 (Ref)
ACC	239 (24.1)	228 (23.0)	0.97 (0.79, 1.20)	0.98 (0.78, 1.23)	126 (24.9)	1.09 (0.85, 1.40)	1.14 (0.87, 1.51)	99 (23.1)	0.97 (0.74, 1.27)	1.01 (0.75, 1.37)
GCC	64(6.5)	86 (8.7)	1.37 (0.97, 1.92)	1.39 (0.96, 2.00)	48 (9.5)	1.55 (1.04, 2.31)	1.64 (1.06, 2.54)	35 (8.2)	1.28 (0.83, 1.97)	1.15 (0.71, 1.85)

Abbreviations: OR, odds ratio; CI, confidence interval.

*Adjusted for age, sex, *H. pylori* infection, smoking status, alcohol consumption, education, and income.

† *IL10*-819 and *IL10*-592 were in complete linkage disequilibrium.

‡ composed of three polymorphic sites; -1082A/G, -819T/C, and -592A/C.

Next, we investigated the effects of *H. pylori* infection status on the association between *IL10* genetic variants and the risk of noncardia gastric cancer (Table 3). *H. pylori-*infected participants had an increased risk of gastric cancer compared to *H. pylori-*negative participants (OR [95% CI]  = 4.30 [3.02−6.11]). The association between *H. pylori* infection and the risk of gastric cancer was stronger among genetic variant carriers, particularly among intestinal-type gastric cancer patients. The interaction between the *IL10*-592 genetic variant and *H. pylori* infection on the risk of intestinal-type gastric cancer was statistically significant (*P* for interaction  = 0.047).

**Table 3:** The Effects of Helicobacter Infection Status on the Association between *IL10* Genetic Variants and the Risk of Noncardia Gastric Cancer, Stratified by Histological Type*.

		All type		Intestinal type		Diffuse type	
		*H. Pylori* (-)	*H. Pylori* (+)	*H. Pylori* (-)	*H. Pylori* (+)	*H. Pylori* (-)	*H. Pylori* (+)
All genotypes	No. of controls/cases	165/50	329/443	165/27	329/225	165/20	329/194
	OR (95% CI)*	1.0 (ref)	4.30(3.02, 6.11)	1.0 (ref)	3.91(2.49, 6.16)	1.0 (ref)	4.84(2.92, 8.03)
***IL10*** **-1082**							
AA	No. of controls/cases	147/42	287/372	147/23	287/185	147/16	287/166
	OR (95% CI)*	1.0 (ref)	4.21(2.89, 6.14)	1.0(ref)	3.64(2.24, 5.91)	1.0(ref)	5.42(3.09, 9.50)
G carrier	No. of controls/cases	18/8	42/71	18/4	42/40	18/4	42/28
	OR (95% CI)*	1.46(0.59, 3.64)	5.59(3.33, 9.41)	1.25(0.38, 4.11)	6.02(3.18, 11.42)	2.13(0.62, 7.27)	5.54(2.69, 11.41)
	*P* for interaction = 0.786			*P* for interaction = 0.705		*P* for interaction = 0.365	
***IL10*** **-592** †							
AA	No. of controls/cases	75/25	172/206	75/15	172/92	75/7	172/95
	OR (95% CI)*	1.0 (ref)	3.21(2.03, 5.44)	1.0 (ref)	2.28(1.22, 4.24)	1.0(ref)	6.48 (2.84, 14.81)
C carrier	No. of controls/cases	90/25	157/237	90/12	157/133	90/13	157/99
	OR (95% CI)*	0.82(0.43, 1.54)	4.25(2.59, 6.95)	0.66(0.29, 1.51)	4.04(2.19, 7.46)	1.80(0.67, 4.81)	7.23(3.17, 16.52)
		*P* for interaction = 0.306		*P* for interaction = 0.047		*P* for interaction = 0.394	

*Adjusted for age, sex, smoking status, alcohol consumption, education, and income.

† *IL10*-819 and *IL10*-592 were in complete linkage disequilibrium.

We also examined the effects of smoking on the association between *IL10* genetic variants and the risk of noncardia gastric cancer (Table 4). The association between current smoking and an increased risk of gastric cancer was stronger amongst *IL10*-1082G carriers and *IL10*-592C carriers. Current smokers that carried the *IL10*-1082G (OR [95% CI]  = 8.72 [3.64−20.91]) and -592C (OR [95% CI]  = 5.40 [2.69−10.84]) alleles had an increased risk of intestinal-type gastric cancer compared to nonsmokers homozygous for *IL10*-1082A and -592A, respectively. However, no statistically significant interactions between *IL10* genetic variants and cigarette smoking on the risk of gastric cancer were observed.

**Table 4:** The Effects of Smoking Status on the Association between *IL10* Genetic Variants and the Risk of Noncardia Gastric Cancer, Stratified by Histological Type*.

			Non-smoker	Past smoker	Current smoker
**All type**	**All genotypes**	No. of controls/cases	208/138	158/108	129/204
		OR (95% CI)*	1.0 (ref)	1.05 (0.66, 1.66)	2.45 (1.58, 3.80)
	***IL10*** **-1082**				
	AA	No. of controls/cases	180/155	140/191	115/170
		OR (95% CI)*	1.0 (ref)	1.00 (0.62, 1.61)	2.23 (1.41, 3.50)
	G carrier	No. of controls/cases	28/28	18/17	14/34
		OR (95% CI)*	1.05 (0.57, 1.93)	1.41 (0.64, 3.12)	4.76 (2.20, 10.30)
		*P* for interaction = 0.323			
	***IL10*** **-592** †				
	AA	No. of controls/cases	105/90	72/46	71/95
		OR (95% CI)*	1.0 (ref)	0.89 (0.49, 1.61)	2.06 (1.20, 3.55)
	C carrier	No. of controls/cases	103/93	86/62	58/109
		OR (95% CI)*	1.00 (0.65, 1.53)	1.16 (0.67, 2.03)	2.88 (1.67, 4.95)
				*P* for interaction = 0.541	
**Intestinal type**	**All genotypes**	No. of controls/cases	208/64	158/71	129/118
		OR (95% CI)*	1.0 (ref)	1.54 (0.87, 2.71)	3.66 (2.12, 6.36)
	***IL10*** **-1082**				
	AA	No. of controls/cases	180/55	140/59	115/95
		OR (95% CI)*	1.0 (ref)	1.43 (0.79, 2.59)	3.18 (1.80, 5.61)
	G carrier	No. of controls/cases	28/9	18/12	14/23
		OR (95% CI)*	1.01 (0.42, 2.41)	2.25 (0.89, 5.66)	8.72(3.64, 20.91)
				*P* for interaction = 0.223	
	***IL10*** **-592** †				
	AA	No. of controls/cases	105/26	72/26	71/55
		OR (95% CI)*	1.0 (ref)	1.29 (0.60, 2.77)	3.42 (1.69, 6.94)
	C carrier	No. of controls/cases	103/38	86/45	58/63
		OR (95% CI)*	1.37 (0.75, 2.51)	2.23 (1.10, 4.51)	5.40 (2.69, 10.84)
				*P* for interaction = 0.867	
**Diffuse type**	**All genotypes**	No. of controls/cases	208/108	158/29	129/77
		OR (95% CI)*	1.0 (ref)	0.64 (0.35, 1.19)	1.82 (1.06, 3.13)
	***IL10*** **-1082**				
	AA	No. of controls/cases	180/91	140/24	115/67
		OR (95% CI)*	1.0 (ref)	0.59 (0.31, 1.12)	1.73 (0.99, 3.02)
	G carrier	No. of controls/cases	28/17	18/5	14/10
		OR (95% CI)*	0.91 (0.45, 1.85)	1.00 (0.32, 3.13)	2.56 (0.94, 7.02)
				*P* for interaction = 0.577	
	***IL10*** **-592** †				
	AA	No. of controls/cases	105/55	72/13	71/34
		OR (95% CI)*	1.0 (ref)	0.50 (0.22, 1.12)	1.44 (0.73, 2.83)
	C carrier	No. of controls/cases	103/53	86/16	58/43
		OR (95% CI)*	0.93 (0.56, 1.54)	0.73 (0.34, 1.56)	2.10(1.09, 4.03)
				*P* for interaction = 0.459	

*The numbers given in the first line for each group are the numbers of controls/cases while the numbers on the second line are the adjusted odds ratios with the 95% confidence intervals in parenthesis. Adjustments were made for age, sex, and *H. pylori* infection status, alcohol consumption, education, and income.

† *IL10*-819 and *IL10*-592 were in complete linkage disequilibrium.

**Figure 1:**
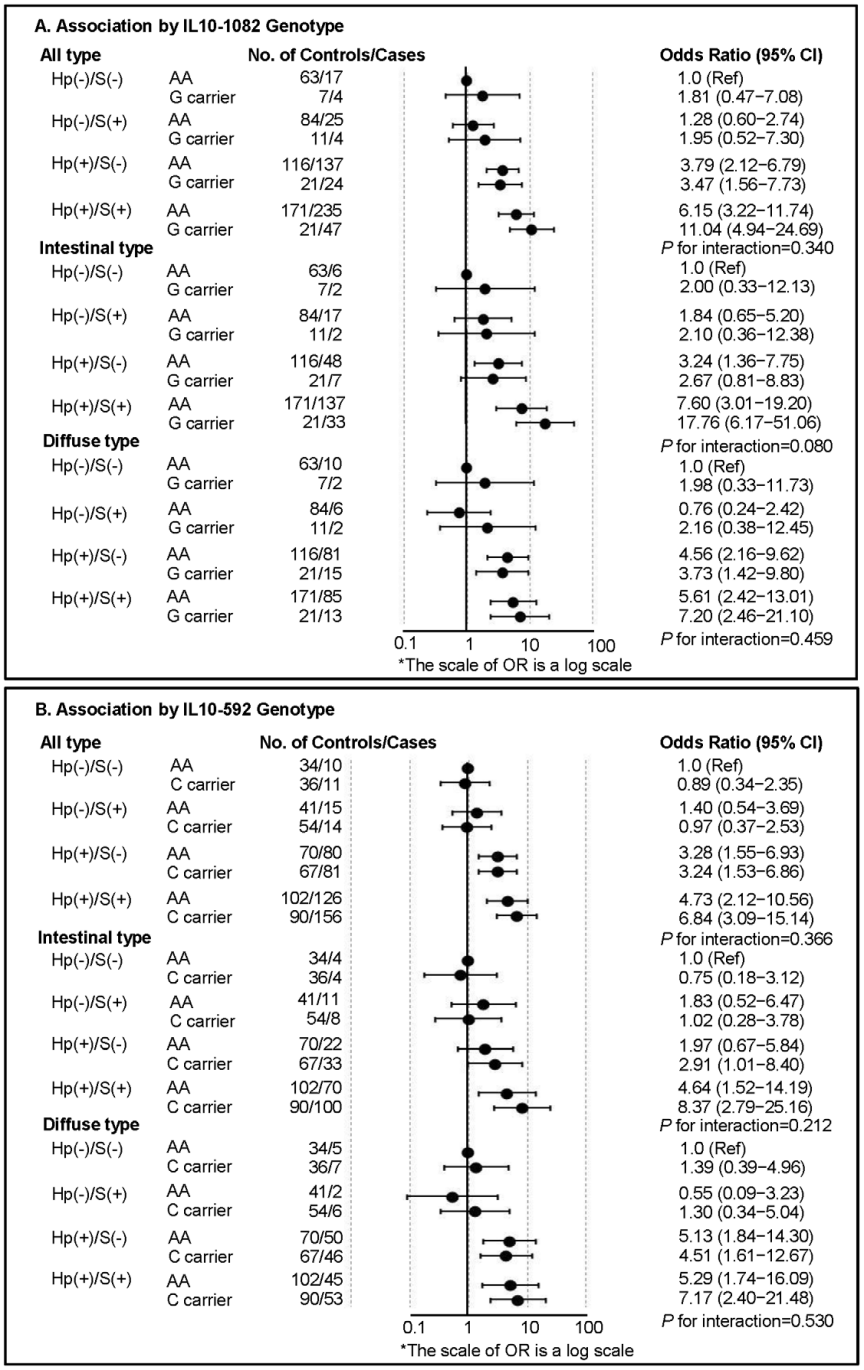
Effects of Environmental Factors on the Association between *IL10* Polymorphisms and the Risk of Noncardia Gastric Cancer. *H. pylori* (Hp) infection and smoking (S) were selected as environmental risk factors, and *IL10* promoter genetic variants (-1082/-819/-592) were investigated. Since *IL10*-819 and -592 were found to be in complete linkage disequilibrium, we presented the data for *IL10*-1082 (A) and -592 (B). Noncardia gastric cancer was stratified by histological type. Multiple logistic regression was adjusted for age, sex, alcohol consumption, education, and income. The significance of the interactions between genetic variants and environmental risk factors was assessed with a likelihood ratio test, which compared a model that included the interaction term with one that only contained the main effects.

To evaluate the interactions between environmental factors and genetic susceptibility, the combined effects of *H. pylori* infection and smoking on the association between *IL10* variants and the risk of noncardia gastric cancer were investigated (Figure 1). Smoking seemed to have no effect in the absence of *H. pylori* infection, but when the data were stratified by genetic variants and histological type, *H. pylori*-positive smokers that carried either the *IL10*-1082G (OR [95% CI]  = 17.76 [6.17−51.06]) or -592C (OR [95% CI]  = 8.37 [2.79−25.16]) allele had an increased risk of intestinal-type gastric cancer compared to *H. pylori-*negative nonsmokers homozygous for *IL10*-1082A allele or -592A allele, respectively. The association between intestinal-type gastric cancer and the combined effects of *H. pylori* infection and smoking was stronger among carriers of the *IL10* genetic variants. The test for interaction between the *IL10*-1082 polymorphisms and the combined effects of *H. pylori* infection and smoking trended towards significance (*P* for interaction  = 0.080).

## Discussion

A number of studies have reported an association between *IL10* promoter polymorphisms (-1082G/-819C/-592C) and the risk of gastric cancer, but these findings have been inconsistent [14], [15], [17]–[19]. In accord with our findings, recent meta-analyses suggested that Asian carriers of the promoter polymorphisms of *IL10* may be associated with an increased risk of gastric cancer [21], [22]. However, some studies, mostly studies conducted in Western populations, have reported a different association [14], [23]. Differences in the number of patients enrolled, study design, patient age at diagnosis, and genotyping methods may contribute to the differences in the results of such studies [9]. In addition, racial differences in the distribution of the *IL10* genotypes may also affect the findings of the studies. Compared to other populations, the frequency of the *IL10* polymorphisms is significantly lower in Asians [24]. This difference may be clinically relevant for susceptibility to gastric cancer and may, in part, explain the different incidences of gastric cancer in Caucasians and Asians [24].

*IL10* genetic variants have been proposed to be associated with higher IL10 production [11]–[13]. IL10 levels are elevated in gastric mucosa infected with *H. pylori* and are higher in patients that have severe chronic inflammation [25]. Furthermore, IL10 mRNA expression and serum levels are elevated in gastric carcinogenesis, particularly in the advanced stage [10], [12], [26], [27]. The role that IL10 plays in carcinogenesis may be due to its ability to act not only as an anti-inflammatory cytokine but also as an immunosuppressant [28]. *H. pylori-*induced IL10 production, while having beneficial effects in terms of limiting the tissue damage caused by inflammation, may also render mucosal immune cells unable to adequately defense against malignant cells [25]. Therefore, the higher levels of IL10 found in the *IL10* genetic variants carriers may partly explain the attenuated immune responses observed during chronic infection. Similarly, the local immunosuppressive cytokine production in chronic gastritis may predispose individuals to the development of gastric carcinogenesis [25]. However, the roles of *IL10* and its polymorphisms in the pathogenesis of gastric cancer require further investigation.

Environmental factors may also play an important role in gastric carcinogenesis [29]. In the present study, the effects of smoking seemed to be confined to *H. pylori*-infected participants, and a synergistic effect between *H. pylori* infection and smoking in terms of increasing the risk of noncardia gastric cancer was observed. This result is in agreement with the findings of several previous studies [30], [31]. Excessive inflammation in response to *H. pylori* infection is associated with an increased vulnerability to gastric carcinogenesis [5]. The synergistic effect of smoking on the risk of developing *H. pylori*-associated cancer may result from various mechanisms that enhance the deleterious state induced by the infection. Smokers have elevated levels of circulating inflammatory mediators, which may exacerbate the detrimental effects of *H. pylori*-associated gastric inflammation [8], [16], [32]. It is also plausible that the gastric mucosal damage caused by *H. pylori*-infection is exacerbated by tobacco carcinogens [33]. The male predominance of gastric cancer, which is related to a 10- to 15-year delay in the development of intestinal-type gastric cancer in females compared to males, may be attributed to the protective role of estrogens against gastric carcinogenesis [34]. However, in light of our findings, the higher rates of *H. pylori* infection and smoking in adult Korean men compared to women may also partly explain the male predominance of gastric cancer in the Korean population [35].

Genetic susceptibility may modify the propensity for gastric carcinogenesis through an alteration of the inflammatory state and may also interact with other risk factors. In the present study, the elevated risk of noncardia gastric cancer in the *IL10* variant carriers was more significant in *H. pylori*-positive subjects who smoked, and these results are similar to the findings of a case-control study in the Taiwanese Chinese population [18]. Another case-control study conducted in Korea reported that a genotype that yields high IL10 production was associated with an increased risk of gastric cancer only within a low soybean intake group, suggesting that the anti-inflammatory effects of soy may counterbalance the pro-carcinogenic effects of the IL10 genetic variants [36]. Taken together, these findings suggest that *H. pylori-*positive smokers with genetic susceptibility to gastric cancer may be an important group to target for prevention and early detection.

Gastric cancer may have different clinicopathological characteristics based on the anatomic site and histological type [6], [37], [38]. In the present study, we investigated only noncardia gastric cancer patients because *H. pylori* is a strong risk factor for noncardia, but not cardia, gastric cancer [39], [40]. We found that *IL10* variant carriers with high levels of inflammation (*H. pylori* infection and smoking) had an increased risk of intestinal-type gastric cancer. Several studies have reported a differential association between genetic variants and the histological types of gastric cancer, but the lack of consistent evidence has hindered the ability to draw any definitive conclusions [9], [23]. Environment and lifestyle are known to affect the development of intestinal-type cancer more than the diffuse-type [41]. It is generally accepted that well-differentiated, intestinal-type gastric cancer develops mostly in older patients through a multistep process that include chronic inflammation, atrophy, and intestinal metaplasia [42]. This notion is supported by our supplemental data that demonstrated that the effects of environmental factors (*H. pylori* infection and smoking) and genetic variants on the risk of advanced atrophy and intestinal metaplasia were similar to those on intestinal-type gastric cancer (Table S1). Therefore, improvements in diet and declines in cigarette smoking and *H. pylori* infections may have contributed to the decrease in intestinal/noncardia type gastric cancer in recent years [38], [41].

This study contains several limitations that should be considered. First, the prevalence of *H. pylori* infection could be underestimated because histological changes in the stomach caused by *H. pylori* might lead to the spontaneous clearance of the bacteria [43]. Additionally, we could not consider *H. pylori* strain variability, which is important because the strains vary in their carcinogenic potential [44]. However, these limitations are likely not considerable because previous data have suggested that the sensitivity and specificity for the *H. pylori* detection method used in the present study were both more than 95% in gastric cancer patients [45] and, in contrast to what is found in Western populations, CagA seroprevalence has been reported to be greater than 95% in infected Koreans [46]. Second, this study is a case-control study. Thus, selection and recall biases may affect the results. Third, the sample size was relatively small, especially for the examination of gene-environment interactions in stratified analyses [47]. Therefore, the near significant interactions between gene and environmental factors or even the null results should be interpreted carefully because of our limited power. Finally, several residual confounding factors may be important to consider. Other genes and environmental factors (e.g., dietary habits) may act either alone or in concert with those studied here in the pathogenesis of gastric cancer. Therefore, studies using a larger number of subjects from different populations are needed to elucidate additional gene-gene and gene-environment interactions in gastric cancer susceptibility [18].

In conclusion, our study found that *IL10* related host susceptibilities may play a role in gastric carcinogenesis at noncardia locations via an interaction with *H. pylori* infection and cigarette smoking. These results support the notion that gastric carcinogenesis is induced in multiple steps that involve both genetic and environmental factors. These findings may be helpful in identifying individuals at an increased risk for developing noncardia gastric cancer.

## Supporting Information

Table S1 **Combined Effects of** ***H. pylori*** **Infection and Smoking on the Association between** ***IL10*** **Genetic Variants and the Risk of Advanced Atrophy and Intestinal Metaplasia in the Antrum and Body of the Stomach**. We evaluated the associations between either intestinal metaplasia or atrophy [advanced stage (grade 2 + grade 3) vs. early stage (grade 0 + grade 1)] and *IL10* SNPs, and the combined effect of *H. pylori* infection and smoking on these associations. The numbers presented on the first line for each group represent the numbers of controls/cases while the numbers on the second line are the adjusted odds ratios with the 95% confidence intervals in parentheses. Adjustments were made for age, sex, alcohol consumption, education, and income. *IL10*-819 and *IL10*-592 were in complete linkage disequilibrium.
(DOC)
