# Supplementary material for: Effects of Interleukin-10 Polymorphisms, Helicobacter pylori Infection, and Smoking on the Risk of Noncardia Gastric Cancer
Source: PLoS One. 2012 Jan 3;7(1):e29643. doi: 10.1371/journal.pone.0029643 (PMC3250465; doi:10.1371/journal.pone.0029643)
Supplement: Table S1 — Combined Effects of H. pylori Infection and Smoking on the Association between IL10 Genetic Variants and the Risk of Advanced Atrophy and Intestinal Metaplasia in the Antrum and Body of the Stomach. We evaluated the associations between either intestinal metaplasia or atrophy [advanced stage (grade 2 + grade 3) vs. early stage (grade 0 + grade 1)] and IL10 SNPs, and the combined effect of H. pylori infection and smoking on these associations. The numbers presented on the first line for each group represent the numbers of controls/cases while the numbers on the second line are the adjusted odds ratios with the 95% confidence intervals in parentheses. Adjustments were made for age, sex, alcohol consumption, education, and income. IL10-819 and IL10-592 were in complete linkage disequilibrium. (DOC) [file pone.0029643.s001.doc]

**Supporting Information**

**Table S1.** Combined Effects of *H. pylori* Infection and Smoking on the Association between *IL10* Genetic Variants and the Risk of Advanced Atrophy and Intestinal Metaplasia in the Antrum and Body of the Stomach^*^

|  | | *H. pylori* (-)/  Smoking (-) | *H. pylori* (-)/  Smoking (+) | *H. pylori* (+)/  Smoking (-) | *H. pylori* (+)/  Smoking (+) |
| --- | --- | --- | --- | --- | --- |
| **Atrophy: Antrum** | |  |  |  |  |
| *Regardless of genotype* | | 66/18 | 75/41 | 79/189 | 124/302 |
|  | | 1.0 (Ref) | 1.70 (0.82, 3.52) | 9.31(5.14, 16.86) | 8.07 (4.22, 15.44) |
| *IL10*-1082 | AA | 61/14 | 69/33 | 62/168 | 112/253 |
|  |  | 1.0 (Ref) | 1.72 (0.78, 3.80) | 11.39 (5.93, 21.89) | 8.57 (4.22, 17.42) |
|  | G carrier | 5/4 | 6/8 | 17/21 | 12/49 |
|  |  | 2.24 (0.51, 9.83) | 4.79 (1.33, 17.27) | 6.36 (2.63, 15.38) | 15.73 (6.28, 39.42) |
| *IL10*-592 | AA | 32/8 | 35/19 | 38/101 | 66/140 |
|  |  | 1.0 (Ref) | 1.65 (0.60, 4.55) | 10.04 (4.31, 23.39) | 6.83 (2.80, 16.70) |
|  | C carrier | 34/10 | 40/22 | 41/88 | 58/162 |
|  |  | 0.97 (0.34, 2.77) | 1.65 (0.61, 4.45) | 8.31 (3.56, 19.40) | 8.96 (3.70, 21.65) |
| **Atrophy: Body** | |  |  |  |  |
| *Regardless of genotype* | | 80/8 | 99/13 | 154/104 | 185/237 |
|  |  | 1.0 (Ref) | 1.33 (0.49, 3.60) | 7.37 (3.37, 16.12) | 13.65 (5.92, 31.48) |
| *IL10*-1082 | AA | 71/6 | 87/12 | 129/94 | 164/200 |
|  |  | 1.0 (Ref) | 1.61 (0.54, 4.84) | 8.76 (3.60, 21.30) | 14.82 (5.78, 37.97) |
|  | G carrier | 9/2 | 12/1 | 25/10 | 21/37 |
|  |  | 1.91 (0.32, 11.32) | 0.91 (0.10, 8.65) | 5.85 (1.88, 18.20) | 22.05 (7.63, 63.68) |
| *IL10*-592 | AA | 93/3 | 43/10 | 81/53 | 92/116 |
|  |  | 1.0 (Ref) | 3.14 (0.76, 13.05) | 9.29 (2.70, 31.96) | 17.66 (4.93, 63.32) |
|  | C carrier | 41/5 | 56/3 | 73/51 | 93/121 |
|  |  | 1.62 (0.36, 7.36) | 0.70 (0.13, 3.86) | 10.10 (2.92, 34.96) | 18.19 (5.12, 64.69) |
| **Intestinal metaplasia: Antrum** | | |  |  |  |
| *Regardless of genotype* | | 76/10 | 91/25 | 192/89 | 219/225 |
|  |  | 1.0 (Ref) | 2.12 (0.89, 5.05) | 3.51 (1.72, 7.17) | 7.99 (3.72, 17.16) |
| *IL10*-1082 | AA | 68/8 | 80/22 | 166/73 | 193/189 |
|  |  | 1.0 (Ref) | 2.22 (0.86, 5.71) | 3.52 (1.59, 7.80) | 7.97 (3.44, 18.48) |
|  | G carrier | 8/2 | 11/3 | 26/16 | 26/36 |
|  |  | 1.65 (0.29, 9.41) | 2.35 (0.51, 10.84) | 5.75 (2.15, 15.36) | 11.98 (4.61, 31.17) |
| *IL10*-592 | AA | 37/4 | 40/14 | 101/41 | 108/106 |
|  |  | 1.0 (Ref) | 3.14 (0.89, 11.13) | 3.61 (1.20, 10.87) | 8.87 (2.84, 27.71) |
|  | C carrier | 39/6 | 51/11 | 91/48 | 111/119 |
|  |  | 1.34 (0.34, 5.20) | 1.97 (0.54, 7.11) | 4.70 (1.57, 14.11) | 9.84 (3.19, 30.41) |
| **Intestinal metaplasia: Body** | | |  |  |  |
| *Regardless of genotype* | | 81/7 | 103/14 | 210/67 | 251/194 |
|  |  | 1.0 (Ref) | 1.69 (0.60, 4.72) | 3.68 (1.60, 8.45) | 9.73 (4.03, 23.53) |
| *IL10*-1082 | AA | 22/5 | 90/13 | 180/58 | 223/162 |
|  |  | 1.0 (Ref) | 2.10 (0.66, 6.63) | 4.35 (1.65, 11.43) | 10.66 (3.67, 29.39) |
|  | G carrier | 9/2 | 13/1 | 30/9 | 28/32 |
|  |  | 2.38 (0.39, 14.50) | 1.24 (0.13, 12.02) | 4.76 (1.44, 15.76) | 18.75 (6.15, 57.16) |
| *IL10*-592 | AA | 39/3 | 47/8 | 107/35 | 130/91 |
|  |  | 1.0 (Ref) | 2.19 (0.50, 9.50) | 4.08 (1.17, 14.24) | 8.95 (2.46, 32.50) |
|  | C carrier | 42/4 | 56/6 | 103/32 | 121/103 |
|  |  | 1.12 (0.23, 5.43) | 1.42 (0.31, 6.44) | 3.74 (1.07, 13.13) | 11.62 (3.23, 41.79) |

^*^ We evaluated the associations between either intestinal metaplasia or atrophy [advanced stage (grade 2 + grade 3) vs. early stage (grade 0 + grade 1)] and *IL10* SNPs, and the combined effect of *H. pylori* infection and smoking on these associations.

The numbers presented on the first line for each group represent the numbers of controls/cases while the numbers on the second line are the adjusted odds ratios with the 95% confidence intervals in parentheses. Adjustments were made for age, sex, alcohol consumption, education, and income.

^†^*IL10*-819 and *IL10*-592 were in complete linkage disequilibrium.
